# Indirect Calorimetry: History, Technology, and Application

**DOI:** 10.3389/fped.2018.00257

**Published:** 2018-09-19

**Authors:** Haifa Mtaweh, Lori Tuira, Alejandro A. Floh, Christopher S. Parshuram

**Affiliations:** ^1^Department of Critical Care Medicine, The Hospital for Sick Children, University Ave, Toronto, ON, Canada; ^2^Department of Clinical Dietetics, The Hospital for Sick Children, University Ave, Toronto, ON, Canada

**Keywords:** calorimetry, indirect calorimetry, energy expenditure, closed-circuit calorimetry, open-circuit calorimetry, critically ill children, energy metabolism

## Abstract

Measurement of energy expenditure is important in order to determine basal metabolic rate and inform energy prescription provided. Indirect calorimetry is the reference standard and clinically recommended means to measure energy expenditure. This article reviews the historical development, technical, and logistic challenges of indirect calorimetry measurement, and provides case examples for practicing clinicians. Formulae to estimate energy expenditure are highly inaccurate and reinforce the role of the indirect calorimetry and the importance of understanding the strength and limitation of the method and its application.

## Introduction

Living organisms are dependent on the constant expenditure of energy-rich adenosine tri-phosphate (ATP) for survival. Energy production is tightly controlled by the organism and is required to sustain cellular homeostasis, organ function, and growth (1–3). The continuous, formation of energy requires a constant supply of substrates: primarily glucose, fatty acids, and oxygen (O_2_). Energy from their combination is used to produce ATP by oxidative phosphorylation, and results in the by-products carbon dioxide (CO_2_) and water (H_2_O) (1, 3).

**Figure 1:**
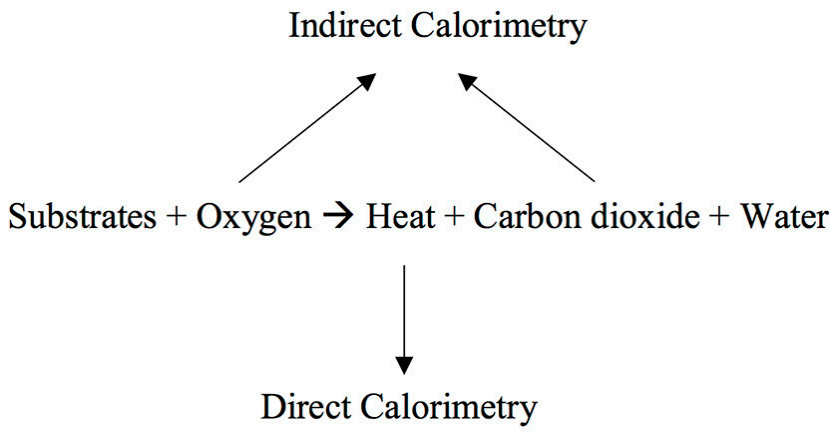
Direct calorimetry measures heat production and indirect calorimetry measures gas exchange: oxygen consumption and carbon dioxide production.

Understanding of cellular metabolism advanced in the Eighteenth century when Lavoisier and Laplace described the measurement of heat exchange (4). In the late Nineteenth century, the first direct calorimeter, the Atwater-Rosa calorimeter, was made. Using the calorimeter scientists were able to demonstrate the law of conservation of energy: the equivalency between fuel (energy) consumed and heat (energy) produced. This work showed that the expenditure of energy by organisms can be measured directly by direct calorimeters or estimated by measuring oxygen consumption (O_2_): indirect calorimetry (4) (Figure 1). Indirect calorimetry is now considered the reference standard for measurement of energy expenditure in critically ill children (5). It is particularly recommended in children with nutritional deficits or derangements due to underlying disorders like cancers or acute diseases like sepsis or multiple trauma that are associated with large inaccuracies in estimation of energy needs, and those who fail attempts at liberation from mechanical ventilation (5–8).

In this review, we summarize the historical context for development of indirect calorimeters, the different systems utilized in energy expenditure measurement along with their limitations, and the clinical considerations required to perform and interpret a measurement of energy expenditure done with indirect calorimetry in critically ill children.

### Historical context

**Figure 2:**
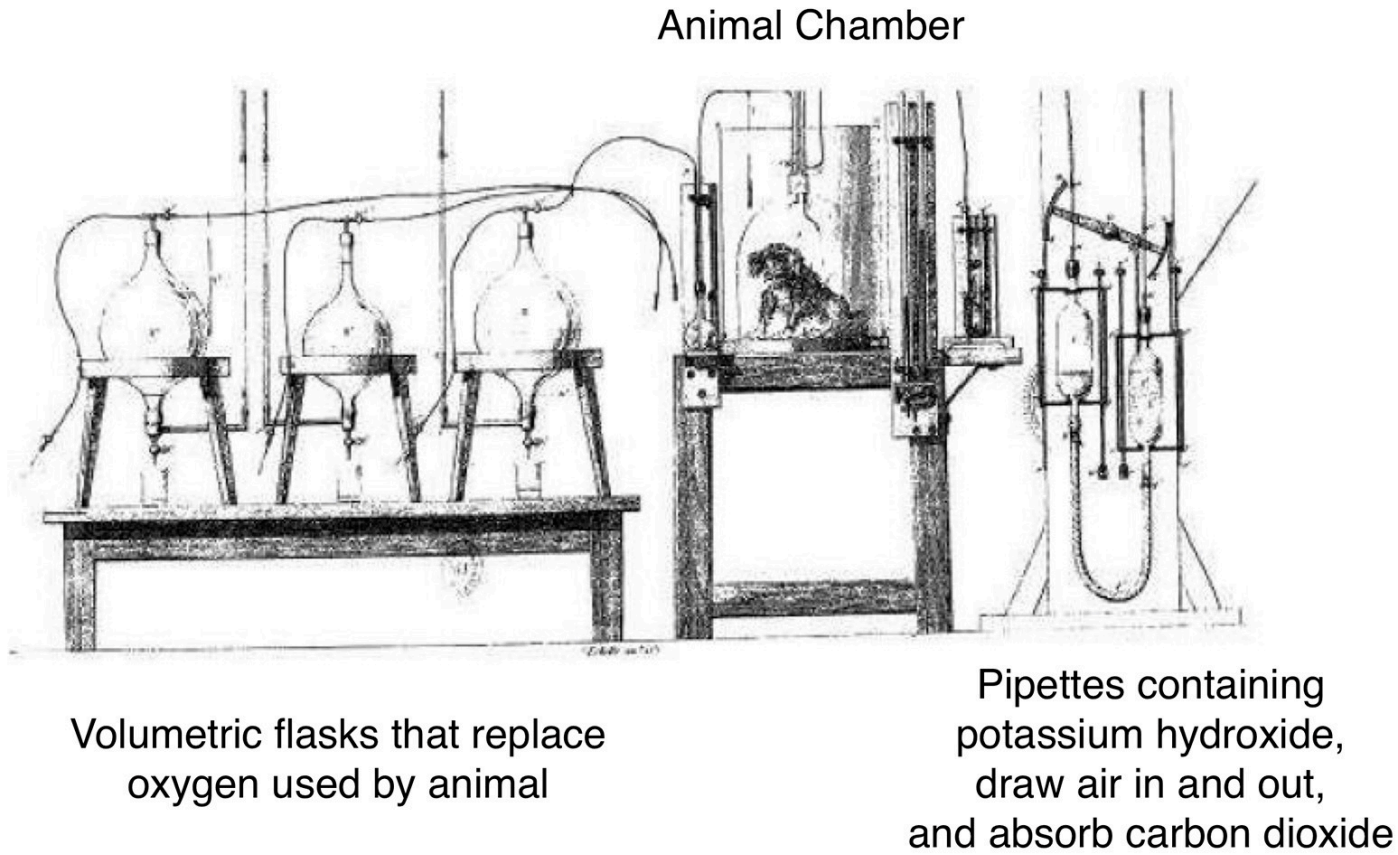
The apparatus of Henri Victor Regnault and Jules Reiset (1849). Adapted from Regnault and Reiset. In this closed loop device, oxygen was supplied to the dog by a tube on the left and carbon dioxide was removed by the tubes on the right. Oxygen was delivered as required to replace that used up by the animal, therefore oxygen consumption was measured by the amount required to maintain constant system pressure. Carbon dioxide was removed by an absorbent and then returned to the respiration chamber to be used over again. Weighing of the absorption vessels allowed measurement of carbon dioxide produced.

Interest in the development of technology to measure energy started in the 1800's when Regnault and Reiset devised a closed-circuit system for measurement of O_2_ consumption, and demonstrated the ratio of CO_2_ produced to O_2_ consumed varied according to the type of food ingested (9, 10) (Figure 2). von Voit and von Pettenkofer determined the proportions of carbon, nitrogen, and O_2_ involved in metabolism in different dietary conditions, and demonstrated that metabolism could be fully interpreted in terms of the oxidation of three types of food substances: protein, fat, and carbohydrate(4, 10). Rubner, in 1894, built a direct calorimeter that measured the heat given by animals based on the temperature change in a surrounding water medium. The animal chamber was also connected to a Pettenkofer respiration chamber for respiratory analysis. Rubner demonstrated complete agreement between the two methods (10). Five years later, the Atwater and Rosa calorimeter employed the open-circuit Pettenkofer system for indirect estimation of heat production, and they coined the term 'respiration calorimeter'. After it had been established that the estimate of heat production from respiratory analysis agreed with the direct measurement of heat, the term indirect calorimeter came to be applied to respiration chambers.

The major development in the indirect measurement of heat production was the development of portable systems. The Tissot spirometer (1904) and the Douglas bag (1911) collected all expired air for analysis and these were limited by the collection vessel size. Therefore, mask methods were developed by (11, 12), and (13) that involved connecting the subject to a static device for measuring change in gas volume. In 1952, Müller and Franz developed an open circuit mask system that could be carried in a bag (10). Since then, the development of indirect calorimetry has allowed its application in the sports and medicine fields.

### Indirect calorimetry assumptions and calculations

Indirect calorimetry methods for energy expenditure measurements are based on the following assumptions (10, 14):

1. Any fuel consumed has an intrinsic energy content that upon metabolic modifications in the living system will result in heat or energy production.
2. The combustion or synthesis of carbohydrate, fat, or protein is the end result of all the biochemical reactions occurring in the body.
3. The oxidation of glucose, fat, or protein results in a substance-specific fixed ratio between the quantities of O_2_ consumed and CO_2_ produced.
4. Loss of substrates is negligible in feces and urine.

The second assumption overlooks the metabolism of minerals that account for 7% of total bodyweight, and the third assumption entails that fat and protein have uniform properties. Notwithstanding the limitations of those assumptions, indirect calorimetry has been found to be consistent and in close agreement with direct calorimetry (10). The methods for estimating the calorific factors and for calculating heat production were developed during the late eighteenth and early Nineteenth centuries and are now mainly of historical and educational interest (15). Newer methods based on algebraic analysis of the calculation procedures are shorter, simpler, and more versatile (10, 16). In open-circuit systems, the following is measured: ventilation rate and the composition of inlet and outlet air, then O_2_ consumption ($\overset{{}^{\circ}}{V}$O_2_), respiratory quotient (RQ), and metabolic rate (Ṁ) are computed.

Gas flowing into the system has a total air flow of V_I_ that has a fraction of inspired O_2_ (F_i_O_2_) and a fraction of inspired CO_2_ (F_i_CO_2_), whereas gas flowing out has a total air flow of V_E_ with an F_e_O_2_ and F_e_CO_2_. Additionally, inspired air has nitrogen therefore

F_i_N_2_ = 1 – F_i_O_2_ – F_i_CO_2_. In the presence of steady state conditions, defined as when sufficient time has elapsed for the outlet gas concentrations to equilibrate with the levels of gas exchange by the subject, the quantity of O_2_ consumed is then VO_2_ = V_I_ x F_i_O_2_ – V_E_ x F_e_O_2_. Since it is technically difficult to measure the small difference in volumes of inspiratory and expiratory air, V_I_ is usually calculated using the Haldane transformation that assumes nitrogen is equal in inspired and expired gas (17, 18), therefore V_I_ x F_i_N_2_ = V_E_ x F_e_N_2_

$$\begin{array}{lll} {\text{V}}_{\text{I}}x\left(1-F_{\text{i}}O_{2}-F_{\text{i}}CO_{2}\right)={\text{V}}_{\text{E}}x\left(1-F_{\text{e}}O_{2}-F_{\text{e}}CO_{2}\right)\\ {\text{V}}_{\text{I}}=V_{\text{E}}x\left[1-\frac{1}{{\text{F}}_{\text{i}}N_{2}\;x\;\left[\left(F_{\text{e}}O_{2}-F_{\text{i}}O_{2}\right)\;+\;\left(F_{\text{e}}CO_{2}-\;F_{\text{e}}O_{2}\right)\right]}\right]\\ \text{And V}{\text{O}}_{2} & = & {\text{V}}_{\text{I}}xF_{\text{i}}O_{2}-V_{\text{E}}xF_{\text{e}}O_{2}\\ = & \left[\left(1-{\text{F}}_{\text{e}}O_{2}-F_{\text{e}}CO_{2}\right)x\left(F_{\text{i}}O_{2}-F_{\text{e}}O_{2}\right)/V_{\text{E}}\right]/\\ \left(1-{\text{F}}_{\text{i}}O_{2}\right) \end{array}$$

As for heat production and the formula used by current indirect calorimeters for energy expenditure calculation, Weir showed that (19):

Ṁ (kcal/min) = [3.941 x $\overset{{}^{\circ}}{V}$O_2_ (liters/ min)] + [1.106 x $\overset{{}^{\circ}}{V}$CO_2_ (liters/min)] – [2.17 x urinary nitrogen (g/day)]

Ṁ (kcal/min) ^*^ 1440 = Ṁ (kcal/day).

### Indirect calorimetry systems

Indirect calorimetry systems estimate respiratory gas exchange as a surrogate for substrates consumed and produced during metabolism. This is done by one of 4 methods: confinement, closed-circuit, total collection, and open-circuit approaches.

1. *Confinement systems:* The rates of change of gas concentrations in a fixed volume is measured for a subject held in a sealed chamber. The limitation is that the measurement has to be restricted to a short period before O_2_ depletion occurs (10).
2. *Closed-circuit systems:* The subject is placed in a closed space with CO_2_ and moisture absorbers, and the quantity of O_2_ used up by the subject is measured. In most versions, only O_2_ consumption is measured (10). The major advantage of this system is that it can be used in patients with high FiO_2_ needs since Haldane transformation is not used (20). The major limitations are the equipment size and poor portability. Closed-circuits could lead to reduction of alveolar ventilation due to increased compressibility of the breathing circuit and may result in increased work of breathing (21, 22).
3. *Total collection systems:* All expired gas by a subject is collected and its volume and chemical composition are measured. Examples of this system is the Douglas bag, which is considered a reference standard in measurement of gas exchange. Limitations are the size of collection bag required and the potential of gas leak from the collection system(23).
4. *Open circuit systems:* The open-circuit chamber method was one of the earliest types of calorimeters and the one used now (10). The subject breathes from the atmosphere and expires into a separate outlet or the subject inspires and expires to a stream of passing air. In both, the flow of air is measured either on the inlet or outlet side of the subject and is either collected periodically or sampled continuously for analysis of gases.

The most commonly used method for gas analysis are the paramagnetic or fuel cell O_2_ sensor and the infrared CO_2_ analyzer (10). This system should not be used in patients requiring FiO_2_ > 0.8 since they utilize the Haldane transformation for VO_2_ calculation, patients with unstable FiO2 within a breath or between breaths, patients with non-reversible endotracheal tube leaks, air leak, or extracorporeal CO_2_ removal (ECMO or dialysis) due to CO_2_ loss that can't be measured, and small patient size (machine variable, lower limit ranges from 5 to 10 kg). All the above affect the applicability of different devices in the pediatric critically ill population (20, 21, 24) (Table 1).

**Table 1:** Considerations for indirect calorimetry.

**Factors**	**Effects**
Gas analyzer precision	Poor precision in VCO_2_, VO_2_, REE
High pressures within the inspiratory limb of the ventilator circuit	Error in gas partial pressures
Ventilator circuit leaks	Falsely reduced alveolar ventilation, VO_2_, VCO_2_, and REE
High inspired oxygen concentrations	VO_2_ approaches infinity when FiO_2_ closer to 1 (Haldane equation)
Instability of the fraction of inspired oxygen during inspiration	Incorrect VO_2_ if FiO_2_ changes between FiO_2_ analysis and expired-gas collection
Meticulous calibration and correct ambient conditions	Poor precision in VCO_2_, VO_2_, REE
Handling of bias flow (flow-by) from the ventilator	If bias flow > 10 L/min, measurement will be invalid (except for Deltatrac)
Dead space created by the ventilator tubing and heat–moisture exchange systems	Results in VCO_2_ changes, hence REE inaccuracy

### Gas analysis and measurement of volume and flow

Gas and flowrate measurements are essential components for accurate indirect calorimetry measurements. The most commonly used gas analyzers are paramagnetic O_2_ analyzers, galvanic O_2_ sensors, and infra-red CO_2_ analyzers (10, 25). While measurement of flow rate can be achieved either by the measurement of the volume of gas expired over a period of time or by the integration of a continuous measurement of rate of flow (Pneumotachometers) by 4 different forms: pressure-differential, turbines, pitot tubes, and hot-wire anemometers (26).

Accurate calibration of gas analyzers is one of the most essential requirements in calorimetry. If an evaluation of the full energy balance of an individual subject, or the metabolic response to some applied experimental treatment is required, then energy production must be measured with the greatest possible accuracy and precision. Additionally, it is important both in calibration and in subsequent measurements that gases entering the analyzers are always conditioned to the same fixed levels of humidity, pressure, flow rate and temperature (25, 26).

### Accuracy and precision of measurements and validity of different systems utilized in pediatric critical care

Accepted standard for levels of precision and accuracy for indirect calorimetry systems are not available. Some authors have suggested that the overall system is dependent on the levels of accuracy and precision of the volume and gas sensors, and to the precision and accuracy of the VO_2_ measurement(27–31). An acceptable level of accuracy for VO_2_ measurement is considered ±4–10% (32, 33).

There is inconsistency in the criteria deciding comparability between a reference technique and new devices for energy expenditure measurements. Other fields in medicine have developed acceptable limits of agreement between devices (34, 35). The most commonly utilized systems in pediatric critical care are summarized below.

1. *Deltatrac II:* This open-circuit calorimeter has two chambers. The first collects the expiratory gas, that is then sampled and the F_e_O_2_ and F_e_CO_2_ analyzed using paramagnetic and infrared analyzers, respectively (36). Next, the expired gas is passed at a constant flow rate (Q) through an air dilution chamber, sampled, and the fraction of CO_2_ is analyzed allowing calculation of the volume of CO_2_ expired: VCO_2_ = F_e_CO_2_ x Q and VO_2_ is calculated through the Haldane transformation^14^. Thus, the minute ventilation of the patient is not directly measured and all gases released at the expiratory port are sampled. The Deltatrac is not affected by the ventilator's bias flow since it lacks a flow measurement technique (17, 34).
2. *Ultima CCM Express:* The CCM Express measures gas exchange through a breath-by-breath technique. This is achieved by the utilizing a pneumotach flowmeter directly connected to the endotracheal tube and gas is collected through a sampling line in the flowmeter. A galvanic fuel cell and an infrared analyzer are utilized for the O_2_ and CO_2_ measurement, respectively. The bias flow provided by the ventilator does not affect measurements since ventilation is measured at the endotracheal tube (37).
3. *Vmax Series:* The Vmax measures mixed expired gas on a breath by breath basis. The O_2_ sensor is an electrochemical fuel cell, and CO_2_ sensor is an infrared one. It utilizes a mass flow sensor connected to exhaust port of the ventilator.

Deltatrac is considered the reference standard after validation studies demonstrated low bias and good precision in comparison to Douglas bag (38). This is the first device that allowed the minute to minute data analysis but has now been discontinued from commercial sale. Takala, Levinson, and colleagues showed that VO_2_ obtained from Deltatrac was consistently slightly higher than those obtained by pulmonary artery catheter or mass spectrometry, however accurate for energy expenditure measurements (18, 39). The VO_2_ discrepancy could be partially related to the lung O_2_ consumption that is not measured by thermodilution (34). Sundström and colleagues compared the Ultima CCM Express to Deltatrac II in mechanically ventilated adult patients and found CCM Express produced 64% higher mean REE values than Deltatrac(37). The VCO_2_ was in particular higher leading to errors in the RQ. No validity studies utilizing the Vmax system were identified in mechanically ventilated patient, however in healthy adults, the Vmax has been shown to have acceptable validity in comparison to the Deltatrac, with limits of agreement of 5 – 10%(40).

### Energy expenditure

Total energy expenditure is a composite of resting energy expenditure (REE) that forms most of the total energy expenditure in critically ill children, thermic effect of feeding (TEF) and activity related energy expenditure (AEE) (41). By convention, indirect calorimetry is used to assess REE while certain conditions are met to mitigate the effect of TEF and AEE. TEF is greater with bolus feeds and less with continuous feeding (2, 3). AEE is lessened by ensuring a resting state for 2 h prior to the measurement (42, 43).

Some reports suggest that energy expenditure measurements should be performed in quiet rooms and mild lighting, but the effect of noise has not been studied (44). Room temperature has been demonstrated to affect energy expenditure in healthy adults (45–47). For accurate resting energy expenditure measurement, attention must be given to ensure steady-state conditions. Steady state is defined by the degree of variation in VO_2_ and VCO_2_ over a set time period. In mechanically ventilated patients, 5-min measurements with 5% coefficient of variation can be equivalent to 30- min measurements with 10% coefficient of variation and both are considered acceptable representations of a steady state (48–50). As for the variability of the energy expenditure measurement during a 24-h period, studies have demonstrated that in critically ill adults, short durations of monitoring are representative of the 24-h and the lack of significant variability between night or daytime measurements (51, 52).

### Considerations for the measurement of energy expenditure by indirect calorimetry

Prior to the start of the measurement, the indirect calorimeter should be calibrated, minimal ventilator circuit or endotracheal tube leaks ensured, FiO2 < 80% and inspired tidal volumes larger than the lower limit set by the manufacturer should be confirmed (Table 1) (14). The patient should be at rest, with last endotracheal tube suction done at least 20 min before the measurement, no ventilatory changes, and minimal change in medications administered for the hour prior. Continuous enteral and parenteral nutrition should be continued. If clinician is interested in TEF in addition to REE, then bolus fed patients should be measured within 1-h of last bolus, otherwise the measurement should occur > 5-h after a feed. The operator should review the results as they are being obtained in order to address measurement issues prior to completion of the study (14, 42, 53, 54).

Results obtained will include measures of $\overset{{}^{\circ}}{V}$O_2_, $\overset{{}^{\circ}}{V}$CO_2_, RQ, REE, and a coefficient of variation of $\overset{{}^{\circ}}{V}$O_2_. Normative values for $\overset{{}^{\circ}}{V}$O_2_ and $\overset{{}^{\circ}}{V}$CO_2_ are reported to be 120 ml/min/m^2^ and 100 ml/min/m^2^ respectively and REE of 25–40 kcal/kg/day (55). An acceptable level of coefficient of variation is < 10% for a 30-min measurement. RQ is the ratio of $\overset{{}^{\circ}}{V}$CO_2_ to $\overset{{}^{\circ}}{V}$O_2_ and used as a marker for substrate utilization. Under standard metabolic conditions with stable respiratory function, the range of RQ in humans is ~0.7–1, with 0.7 representing predominantly fat utilization, 0.8 for mixed diet, and 1 for carbohydrates (20, 53, 56, 57). A summary of causes of altered results are summarized in Table 2.

**Table 2:** Result interpretation.

**Results**	**Cause**
Elevated VCO_2_ and RQ	Metabolic acidosis Hyperventilation Hypermetabolism Excessive carbohydrate intake
Decreased VCO_2_ and RQ	Metabolic alkalosis Hypometabolism Starvation/ketosis Hypoventilation Gluconeogenesis Underfeeding Oxidation of ethanol Air leak
Elevated VO_2_	Sepsis Hypermetabolism Hyperthermia Blood transfusions Shivering/agitation/excessive movement Increased minute ventilation Hemodialysis (within 4 h of treatment) Overfeeding
Decreased VO_2_	Hypothermia Hypothyroidism Paralysis Heavy sedation Fasting/starvation Advanced age General anesthesia Coma/deep sleep

*VCO_2_, Carbon dioxide production; RQ, Respiratory quotient; VO_2_, Oxygen consumption*.

### Indications and limitations for indirect calorimetry in critically ILL children

All critically ill patients are susceptible to under and over nutrition, hence measurement of energy expenditure and the titration of intake based on the results is recommended (5). However, indirect calorimetry has suffered from limited spread that could be related to the high monetary cost to purchase and maintain the equipment, and the time cost to perform the measurement (58). This has led nutritional societies to develop recommendations for certain patient populations where measurement of energy expenditure should be performed: (a) clinical conditions that significantly alter REE; (b) when patients fail to respond to presumed adequate nutrition support; and (c) in order to individualize the nutrition support in the ICU. More particular examples include patients with altered body composition, continued weight loss in face of “adequate” nutrition, persistent inflammatory state (for example severe burns, trauma, prolonged septic states), and difficulty in mechanical ventilation weaning (5, 58, 58–60). This recommendation aims to assist clinicians in prioritizing a scarce resource, however is inconsistent with the statements of both ASPEN and ESPEN that indirect calorimetry is the reference standard for energy expenditure measurement (5, 59). Energy expenditure measurement has been shown to affect patient management, particularly resulting in a change in the nutritional prescription in 75–80% of patients in two recent studies, but how this impacts patients' outcomes remains unclear (61, 62). Moreover, no prospective pediatric trials have been conducted to compare the effect of titrated nutritional delivery to energy expenditure and its effects on outcome. In the latest and largest pediatric randomized trial in nutrition (PePANIC), formulas were used to estimate caloric needs of patients, therefore the effects of their intervention is difficult to interpret since the patient population could have been under- or over-fed (63).

### Alternatives to indirect calorimetry in critically ILL

Predictive equations that estimate energy expenditure have been developed and are used when access to indirect calorimetry is not available. Those developed to estimate energy expenditure in healthy children and extrapolated to use in the critically ill have been reported to be inaccurate in different populations, and their detailed review is out of the scope of this manuscript (6, 64–67). However, two equations have been derived for use in critically ill children: White and Meyer (68, 69). A recent validation study compared energy expenditure predicted by those two equations to measured energy expenditure by indirect calorimetry in a patient population similar to the one the equations were derived from and found errors of −20 to +50% in both (70). This suggests that clinical use is associated with significant errors in estimation of energy requirements in critically ill children. This inaccuracy could be related to: limitations of equation development methods/techniques, narrow range of children studied (disease/conditions/treatments), evaluation of modest numbers of children in datasets used for initial development and validation, and the numbers studied of the above with reduction in power to exclude important effects.

### Case and example of interpretation of results

A 16-year-old male weighing 90 kg, admitted after a subarachnoid hemorrhage. He was mechanically ventilated, pain controlled with a morphine infusion, sedated with intermittent diazepam, and muscle relaxed with a cisatracurium infusion. Patient had an external ventricular drain for intracranial pressure monitoring and cerebrospinal fluid drainage. An indirect calorimetry measurement was performed on day 4 after admission. The patient was normothermic, with no seizures on electroencephalogram. He was on continuous feeds with a nasogastric tube with total caloric intake of 1,500 Kcal/day and protein of 1.5 g/kg/day.

The patient had an endotracheal tube (ETT) suction done 1 h ago, and he had no ETT leak. The coefficient of variation in the first 5 min was 4%. The measurement was made over a period of 30 min. At the conclusion of the test, the results were: VO_2_ = 0.49 L/min, VCO_2_ = 0.35 L/min, RQ = 0.71, measured REE = 3336 Kcal/day.

1. Was the measurement performed at an appropriate time based on the data provided? Yes, the patient was continuously fed, on stable doses of medication, afebrile, had no seizures, and no ETT leak.
2. Can this measurement be accepted as a reliable one? Yes, the coefficient of variation is within accepted limits.
3. What does an RQ of 0.71 represent? This is an RQ within the acceptable normal range for substrate utilization and suggests predominantly fat utilization. This RQ indicates that the patient might require additional caloric intake in the form of carbohydrates to mitigate the lipid metabolism. Causes for abnormally low or high RQ are in Table 2.
4. What does an REE of 3336 Kcal/day mean? There are no reported normal REE ranges according to age or body size in critically ill children (5). Adult guidelines suggest that 25–30 Kcal/kg/day is an acceptable energy target (71). Our patient's REE is 37 Kcal/kg/day and the clinician should adjust the nutritional prescription to provide the current REE due to the significant morbidities associated with both under- and over-feeding (63, 72–80). REE does not provide the clinician with insight in regards to protein turnover and needs, a 24 h urinary nitrogen would be helpful in that case. An acceptable rule of thumb is a protein intake of 1.5–2 g/kg/day based on adult and pediatric nutrition guidelines (5, 71).

### Future directions

Technologic advancement has allowed for the portable measurement of energy expenditure at the bedside via indirect calorimeters. Recently, the incorporation of energy expenditure measurement modules into ventilators has expanded the reach and utility of this tool, but clinicians need to be aware that these new generation ventilators have not been validated against existing reference standards for energy expenditure measurement in critically ill children. The nutritional community must perform validation studies prior to incorporating those measurements into daily practice. Future prospects could include the return of Douglas bag measurements in critically ill patients since these were considered “simple” to use with a lower monetary and time cost. Additional opportunities that have not been explored in this field include utilization of the electronic medical record data and inclusion of artificial intelligence algorithms that would generate energy expenditure as a continuous vital sign. Prior to the development of algorithms though, well designed studies that attempt to determine the effect of different clinical factors and interventions on energy expenditure are required.

## Conclusion

Indirect calorimetry is the reference standard for measurement of energy expenditure in the critically ill. The technology has limitations that should be understood by the clinician performing and interpreting the measurement. Current equations derived for use in critically ill children are not valid and their use should be avoided. The large discrepancies between the estimations and measurement of energy expenditure influences the nutritional management and could impact the outcomes of critically ill children.

## Author contributions

All authors listed have made a substantial, direct, and intellectual contribution to the work and approved it for publication.

### Conflict of interest statement

The authors declare that the research was conducted in the absence of any commercial or financial relationships that could be construed as a potential conflict of interest.
